# Determinants of the utilization of allergy management measures among hay fever sufferers: a theory-based cross-sectional study

**DOI:** 10.1186/s12889-020-09959-w

**Published:** 2020-12-07

**Authors:** Anna Muzalyova, Jens O. Brunner

**Affiliations:** Chair of Health Care Operations/ Health Information Management, UNIKA-T, University of Augsburg, Universitätsstraße 16, 86159 Augsburg, Germany

**Keywords:** Hay fever management, Utilization of health measures, Protection motivation theory, Transtheoretical model, Threat appraisal

## Abstract

**Background:**

The quality of life of chronically ill individuals, such as hay fever sufferers, is significantly dependent on their health behavior. This survey aimed to explain the health-related behavior of allergic individuals using the protection motivation theory (PMT) and the transtheoretical model (TTM).

**Methods:**

The influencing variables stated by PMT were operationalized based on data from semistructured pilot interviews and a pretest with 12 individuals from the target population. The final questionnaire inquired perceived seriousness and severity of hay fever, response efficacy, response costs, self-efficacy, and the use of various hay fever management measures in relation to the TTM stages. Multivariate logistic regression was performed to investigate the relationships between the PMT constructs and the examined health behavior.

**Results:**

A total of 569 allergic individuals completed the online questionnaire. Only 33.26% of allergic individuals were in the maintenance stage for treatment under medical supervision, and almost 60% preferred hay fever self-management. A total of 67.56% had a well-established habit of taking anti-allergic medication, but only 25.31% had undergone specific immunotherapy. The likelihood of seeking medical supervision was positively influenced by perceived severity (OR = 1.35, 95% CI: 1.02–1.81), perceived seriousness (OR = 2.12, 95% CI: 1.56–2.89), and self-efficacy (OR = 4.52, 95% CI: 3.11–6.65). The perceived severity of symptoms predicted the practice of hay fever self-management (OR = 1.60, 95% CI: 1.21–2.11), as well as anti-allergic medication intake (OR = 1.65, 95% CI: 1.16–2.35). The latter measure was also positively influenced by self-efficacy (OR = 1.52, 95% CI: 1.01–2.28) and hay fever self-management (OR = 4.76, 95% CI: 2.67–7.49). Undergoing specific immunotherapy was significantly predicted only by medical supervision (OR = 9.80, 95% CI: 8.16–13.80). Allergen avoidance was a strategy used by allergic individuals who preferred hay fever self-management (OR = 2.56, 95% CI: 1.87–3.52) and experienced notable symptom severity (OR = 2.12, 95% CI: 1.60–2.81).

**Conclusion:**

Educational interventions that increase the awareness of health risks associated with inadequate hay fever management and measures to increase self-efficacy might be beneficial for the promotion of appropriate hay fever management among allergic individuals.

**Supplementary Information:**

The online version contains supplementary material available at 10.1186/s12889-020-09959-w.

## Background

Quality of health and quality of life are essentially dependent on an individual’s lifestyle and health habits. By managing their health behavior, individuals can significantly improve their well-being and live longer and healthier lives. This consideration is especially important for individuals suffering from chronic health conditions, which significantly influence their health-related quality of life. Allergic rhinitis, also called hay fever, is considered by the World Allergy Organization to be one of the most prevalent chronic diseases of the respiratory tract [1]. In Europe, the prevalence of hay fever stagnates on a high level ranging from 13 to 25% among industrialized countries [2] and continues to grow in its prevalence and severity [3], especially in children [4]. Hay fever, as a chronic disease, impairs the everyday activities, sleep quality, and workplace productivity of its sufferers [5, 6] and lowers their perceived quality of life [7–10]. Hay fever sufferers have several options to control the severity of allergic symptoms, such as the intake of antihistamines, specific immunotherapy, or allergen avoidance [11]. Despite the benefits of allergy management through such practices, approximately 70% of hay fever sufferers do not treat their disease properly [12] or fail to meet current allergy management recommendations. Nevertheless, one of four allergic individuals undergoes no treatment despite having symptoms [13]. Therefore, the question of how hay fever sufferers can be motivated to manage their disease remains a crucial research direction.

The scientific community has mostly focused on the supply side of the problem of hay fever and the development of new medications and therapies to be offered to disease sufferers. However, it is reasonable to examine the other side of the problem and shift the research focus on the demand side [14]. This approach can help to promote effective self-management and health habits that keep people healthy across their lifespan. In general, theories and models help predict and explain health behavior in various health-related contexts [15, 16]. Theories and models of health behavior can be classified as motivational, behavioral inaction, and multistage behavior change [17]. Motivational theories propose continuous models to predict health behavior at a single point in time or identify the influencing factors of health-related behavior. Behavioral inaction theories are intended to close the gap between motivation and actual behavior. Multistage models assume that individual progress through various stages in the execution of the desired behavior that ranges from the intention to engage in the target behavior to the maintenance of the target behavior [18]. Multistage models highlight the dynamic nature of behavior change based on the assumption that individuals at different stages think and behave in qualitatively different ways [19].

Protection motivation theory (PMT) is a prominent example of a motivational theory of health behavior first described by Rogers in 1975 [20]. There is a large body of research that has used PMT to explain health behavior in different health-related contexts, including self-reported adherence to corticosteroid medication among asthma patients [21], self-reported adherence to weight loss recommendations [22], self-reported adherence to therapy among people with coronary heart disease [23], the promotion of exercise and healthy dietary behavior [24], and nonpharmaceutical protective behavior during influenza outbreaks [25]. Stage-based approaches to behavior change, such as the transtheoretical model (TTM), have received widespread scientific approval. There is a large body of research that has applied the TTM in a variety of health behavior contexts, including asthma self-management [26], physical activity [27], obesity prevention [28], sun protection [29], and smoking cessation [30].

The main research objective of the present study was to explain the health-related behavior of hay fever sufferers based on protection motivation theory, as this theory was considered to be the most suitable for the specific features of hay fever. The stages of behavior change outlined in the TTM were employed in this study to provide a more detailed and comprehensive differentiation of *actors* and *nonactors* regarding the use of different allergy management measures among allergic individuals. To our knowledge, neither the PMT nor the TTM has been used in previous research to assess the behavior of allergic individuals regarding their hay fever management. The influencing variables corresponding to the PMT constructs were expected to explain the motivation of allergic individuals to undertake or forego particular health-related measures. Therefore, the research question examined in the present survey was formulated as follows:

How do the PMT constructs influence the utilization of different hay fever management measures among allergic individuals?

## Methods

### Operationalization of the PMT constructs

The operationalization of the PMT constructs was carried out in two stages: pilot semistructured interviews and a pretest with a sample of 12 individuals from the target population. The participants in the preliminary analysis were recruited from a pool of allergic individuals who had participated in our previous research [12]. The interviews aimed to identify the participants’ salient perceptions of the health threat caused by hay fever as well as the perceived reasonability and efficacy of the possible health-related measures aimed at reducing allergic symptoms. The main objective of the pretest using draft versions of the PMT scales was to test respondents’ comprehension of the scales and determine appropriate wording for the statements.

The interviews consisted of open-ended questions regarding the PMT constructs and closed-ended questions regarding the utilization of various hay fever management measures. Each recruited allergic individual completed a semistructured interview covering topics presented in the following section.

#### Threat appraisal

According to PMT, protection motivation results from an evaluation process consisting of threat appraisal and response appraisal. The appraisal of the threat posed by a disease consists of two major components: perceived symptom *severity* and perceived *vulnerability*, i.e., the likelihood of the occurrence of the disease [18]. Perceived *vulnerability* was operationalized as perceived *seriousness* of hay fever since allergic individuals already had the disease of interest, and thus, it would not be relevant for them to appraise their likelihood of getting hay fever. There are some interesting insights in our previous research suggesting a crucial impact of the perceived *seriousness* of hay fever on its management [12], making this adaptation reasonable. Along with the study of Bennett et al. (1998) investigating adherence to preventive asthma medication, in the present study the perceived *seriousness* of hay fever was inquired as the perceived chronicity of hay fever [21]. Also, the inquiries of threat appraisal focused on the perceived need for hay fever treatment and its advantages and disadvantages. The perceived *severity* of hay fever was assessed based on the negative effects of allergic symptoms on various dimensions of everyday life, with a focus on social functioning, school or workplace productivity, and quality of sleep when symptomatic.

#### Response appraisal

Response appraisal describes the assessment of the efficacy or value of certain health behavior in reducing the threat caused by a disease and consists of three components: *response efficacy, response costs*, and *self-efficacy*. *Response efficacy* measures the expected benefit of the target behavior in preventing the disease or its harmful influence on health-related well-being. *Response costs* comprise the expected physical, psychological, financial, and other efforts involved in the target behavior. *Self-efficacy* describes the extent to which an individual feels capable of performing the target behavior [18]. To initiate a particular health behavior, the utility of *response efficacy* and *self-efficacy* has to outweigh the *response costs* of the target behavior [16].

*Response efficacy* was operationalized as the perceived or possible positive effects of known hay fever measures on health-related well-being when symptomatic. The open-ended questions concerning *response efficacy* were designed to address the dimensions that allergic individuals mentioned being negatively affected by allergic symptoms in the previous part of the interview. *Response costs* were estimated as the barriers preventing allergic individuals from taking action or the inconvenience arising from the need to take hay fever management measures consistently and regularly. *Self-efficacy* was assessed by questions regarding the individual’s perceived ability to positively influence his or her well-being when symptomatic. Another dimension of *self-efficacy* was the individual’s perceived capability of carrying out health-related measures regularly and consistently.

#### Pretest

Following the interviews, draft versions of the PMT scale were administered to the recruited interviewees. We developed the scales based on an extensive literature review [21, 23, 25, 31, 32], and our previous study investigating health-related impairment caused by allergic symptoms and salient beliefs regarding allergy management [12]. All items assessing the PMT constructs were drafted as closed-ended questions consisting of belief statements followed by a five-point Likert scale (1 = totally disagree, 2 = somewhat disagree, 3 = neutral, 4 = somewhat agree, 5 = totally agree). Allergic individuals were asked to assess the applicability of the statements to themselves. Furthermore, we have encouraged study participants to give general feedback on the questionnaire and to indicate items that were not clearly formulated or were difficult to understand.

Based on the insights obtained in the interviews, additional items with belief statements that had not been previously considered were added to the original drafts of the PMT scales. The less important beliefs corresponding to each PMT component were excluded from the final questionnaire. Additionally, the wording of some items was changed to be more explicit and easy to understand. The semistructured interview guide and the final questionnaire can be found in supplementary materials.

### Operationalization of the allergy management across the TTM stages

Allergy management was assessed with five items specifying five possible allergy management measures: treatment under medical supervision, hay fever self-management, anti-allergic medication, specific immunotherapy, and allergen avoidance. The respondents were asked to indicate their current stage in the utilization of each hay fever management measure. The possible answers to the statements, e.g. “I take anti-allergic medication” were “no, and I do not intend to” (pre-contemplation); “no, but I might think about it” (contemplation); “no, but I strongly intend to do so” (preparation); “yes, but I only started doing it recently” (action); and “yes, and I have been doing it for a long time” (maintenance). Therefore, the use of the TTM allowed us to evaluate both intention and action regarding the target behavior.

Based on the insights from the pretest and pilot interviews the final questionnaire consisted of 33 items related to the PMT constructs, five items on hay fever management, and several items inquiring about the allergy characteristics of survey participants.

### Participants and procedure

This cross-sectional study was conducted between the 9th and 30th of September 2019 using an online survey tool. The start date of the study was intentionally chosen to avoid high pollen load and thus to increase the likelihood of study participants being asymptomatic at the time of the study**.** The study target group was defined as hay fever sufferers who are sensitized exclusively to airborne pollen. Consequently, only participants who were allergic to airborne pollen and did not have additional perennial allergies to dust mites or animal dander were included in the study. All study participants who did not meet the defined inclusion criteria were excluded from the data analysis. The inclusion of the participants in the study was based on self-reported information on pollen sensitization.

The questionnaires were distributed to all participants who were recruited during the previous study conducted in June and July 2016 to collect data on the health behavior of hay fever sufferers. Additionally, we invited all matriculated students of the University of Augsburg as well as all university personnel who were allergic to airborne pollen to participate in the present survey. A total of 805 invited allergic individuals accessed the online questionnaire, and 569 (70.7%) completed it. Eight study participants were excluded since they did not fulfill the inclusion criteria due to an additional sensitization to dust mites (*n* = 7) or animal dander (*n* = 1). Consequently, the remaining 561 participants were found eligible, and their responses were used for data analysis.

The calculation of the minimal sample size was carried out based on Riley et al. (2019). Anticipating a Van Houwelingen shrinkage factor of 0.90, and adjusted Cox-Snell R^2^ of 0.18 with up to 11 parameters to be estimated, the minimum sample size accounts for 494 participants [33]. Therefore, the sample size of 561 participants is appropriate for the given research setting.

#### Data analysis

The survey results were analyzed using SPSS 25 (Statistical Package for the Social Sciences; SPSS Inc., Chicago, IL, USA) and AMOS SPSS 25. The majority of eligible study participants (85.56%, *n* = 480) answered all items, and no item had more than 3.21% (*n* = 18) missing values. The missing values for the items on PMT components were imputed using SPSS MVA regression with gender, allergy characteristics, and responses to the complete PMT subscale items as predictors. In line with the multiple imputation routines, a total of *m =* 50 different datasets were imputed to assure the appropriate statistical power of the inferencestatistical analysis of the data [34].

Construct validity of the developed measurement instrument was evaluated through explanatory factor analysis using oblimin rotation. The number of underlying factors was determined using parallel analysis [35]. For all inquired latent variables, reliability in terms of Cronbach’s alpha, composite reliability (CR), and average variance extracted (AVE) were examined to assure the appropriateness of the measurement model. The overall fit of the proposed measurement model was evaluated using a confirmatory factor. In the present study *χ*^*2*^*/df* ratio, Tucker-Lewis index (TLI), Comparative Fit Index (CFI), and Root Mean Square Error of Approximation (RMSEA) are reported. The *χ*^*2*^*/df* ratio of less than 2.0, TLI and CFI greater than 0.9, and RMSEA < 0.10 are considered to indicate an appropriate model fit [36].

The measurement scales for each PMT construct were averaged across the items to be used in the logistic regression as independent variables. Frequencies were used to describe the utilization rates of different hay fever management measures. Multivariate logistic regression was performed to investigate the relationships between the PMT constructs used as independent variables and the aggregated TTM stage variables used as dependent variables. Additionally, the TTM stage variables were stepwise imputed in the model as independent variables to investigate their interdependencies. Statistical significance was considered at the 0.95 level (*p* < 0.05). To address the current criticism of frequentist methods and *p*-values specifically, we used multiple tests. Consequently, all *p*-values given in this paper were adjusted for multiplicity using the Benjamini-Yekutieli step-up procedure [37].

## Results

### Descriptive results

Females constituted 61.85% (*n* = 347) of the total study sample. The study participants had exhibited allergic symptoms for 14.59 (SD = 9.80) years. Information on the use of various hay fever management measures is outlined in Table 1. Only 33.16% (*n* = 186) of the study sample was in the maintenance stage for allergy treatment under medical supervision, whereas almost 60 % (*n* = 333) were in the maintenance stage for allergy self-management. Approximately 30 % (*n* = 171) claimed that they were not being treated under medical supervision and that they did not intend to do so (pre-contemplation stage). Those who had a well-established habit of taking anti-allergic medication accounted for 67.56% (*n* = 379). Only 25.31% (*n* = 142) of all individuals were in the maintenance stage of specific immunotherapy, but another 4.81% (*n* = 27) were in the action stage, having started it recently. A total of 27.09% (*n* = 152) of the survey participants stated that they were thinking about the possibility of utilizing specific immunotherapy but were not currently undergoing it (contemplation stage). Almost half (*n* = 277) of all participants were in the maintenance stage for allergen avoidance measures, whereas approximately one-fifth had completely stopped practicing this allergy management measure.

**Table 1:** Utilization of different allergy management measures expressed as TTM stages (in %)

	Early-stage (not taking action)				Late-stage (taking action)		
	Pre-contemplation	Contemplation	Preparation	*Σ*	Action	Maintenance	*Σ*
Medical supervision	30.48	22.82	6.42	*59.72*	7.13	33.15	*40.28*
Self-management	18.18	7.49	2.50	*28.17*	12.48	59.35	*71.83*
Anti-allergic medication	10.70	7.13	1.43	*19.26*	13.19	67.55	*80.74*
Specific immunotherapy	37.62	27.09	5.17	*69.88*	4.81	25.31	*30.12*
Allergen avoidance	21.21	11.41	4.28	*36.90*	13.73	49.37	*63.10*

Due to a small size resulting in a small number of survey participants at the contemplation and preparation stages, the TTM stage variables were aggregated as dichotomous, categorical variables to differentiate between early-stage and late-stage of action regarding five examined allergy management measures. Following Prochaska et al. (1992), the early-stage included pre-contemplation, contemplation, and preparation stages of behavior change, and the late-stage included action and maintenance [38]. Therefore, participants in the early stages were not utilizing particular hay fever management measures, including those reporting total resignation, contemplation, or intention of a target behavior. Participants in the late stages included allergic individuals who had utilized particular hay fever management measures recently or for a longer time. Consequently, study participants in the two categories can be referred to as *nonactors* and *actors.*

### Assessment of the measurement model

In the first stage of the data analysis, the reliability and validity of the constructed PMT scales were assessed. The parallel analysis has determined six factors explaining 52.379% of the total variance to be extracted (Table 2).

**Table 2:** Results of the parallel analysis

	Parallel analysis		Explorative factor analysis		
Factor	Means	Percentile	Eigenvalue	Explained variance (%)	Cumulated variance (%)
1	1.485	1.534	5.979	18.119	18.119
2	1.422	1.459	4.876	14.776	32.895
3	1.373	1.411	2.153	6.526	39.420
4	1.336	1.370	1.593	4.829	44.249
5	1.296	1.332	1.369	4.148	48.397
6	1.266	1.291	1.314	3.982	52.379
7	1.235	1.258	1.112	3.371	55.751

The items forming each factor were identified after oblimin rotation was applied. Analysis of the rotated factor matrix reported in Table 3 showed that the latent construct *self-efficacy* consisted of 2 underlying subconstructs. To maintain the unidimensionality of the *self-efficacy* scale, the items forming the factor with lower eigenvalues and the proportion of explained variance were excluded from the further analysis. Additionally, three items of the latent construct *seriousness* were excluded due to their low factor loadings. Furthermore, one item each of the *response efficacy* and *self-efficacy* scales was omitted due to low factor loading, and one item of the *response costs* scale was excluded due to a cross-loading on the *response efficacy* scale. Consequently, the final measurement model consisted of 22 items reflecting five PMT variables. The Cronbach’s alpha (Table 3) of the examined PMT scales was above the value of 0.7 indicating appropriate reliability for all latent constructs, and the CR and AVE of each scale were above the recommended values of 0.7 and 0.5, respectively [39]*.* Overall, the presented results allow the conclusion that all captured latent constructs have the appropriate reliability and convergent validity.

**Table 3:** Rotated factor loading matrix (oblimin rotation)

	Factors					
	1	2	3	4	5	6
**Seriousness**						
*Cronbach’s Alpha* = 0.709, CR = 0.756, AVE = 0.509, Mean (SD) = 3.53(0.786)						
Hay fever is a disease	0.179	− 0.0180	0.116	**0.751**	− 0.022	0.012
Hay fever is a chronic health condition [Ex]	0.208	0.055	0.213	**0.591**	−0.073	0.180
Hay fever is a serious health condition	0.421	0.132	0.116	**0.649**	− 0.064	0.183
Hay fever is a health condition that has to be treated [Ex]	0.355	0.208	0.023	**0.537**	0.030	0.358
I see my hay fever as a long-term health condition	0.389	0.099	0.142	**0.736**	0.011	0.007
Hay fever influences an individual’s well-being [Ex]	0.198	0.176	−0.007	**0.344**	−0.041	0.006
**Severity**						
*Cronbach’s Alpha* = 0.866, CR = 0.900, AVE = 0.600, Mean (SD) = 3.62(0.848)						
…I feel physically burdened	**0.812**	0.066	0.225	0.325	−0.089	0.070
…I feel limited in my social life	**0.772**	−0.018	0.256	0.387	−0.115	0.059
…I feel limited in my spare time	**0.815**	−0.011	0.217	0.335	−0.077	0.024
…my sleep quality is burdened	**0.662**	−0.014	0.233	0.262	−0.219	0.061
…my overall well-being is burdened	**0.799**	−0.006	0.146	0.304	−0.097	0.075
…I experience a loss of productivity	**0.773**	0.085	0.082	0.288	−0.051	0.066
**Response efficacy**						
*Cronbach’s Alpha* = 0.817, CR = 0.856, AVE = 0.598, Mean (SD) = 3.91 (0.714)						
…weakens symptom severity	−0.032	**0.752**	−0.203	− 0.056	0.341	0.175
…helps better control allergic symptoms	−0.034	**0.774**	−0.029	0.283	0.083	0.261
…helps manage everyday routines	0.052	**0.762**	−0.111	0.222	0.252	0.190
…improve overall well-being	0.017	**0.804**	−0.249	0.001	0.291	0.232
…helps staying productive during the pollen season [Ex]	0.041	**0.553**	−0.180	0.083	0.337	0.168
**Response costs**						
*Cronbach’s Alpha* = 0.789, CR = 0.770, AVE = 0.527, Mean (SD) = 2.95(0.882)						
…is time consuming	0.036	−0.016	**0.739**	0.150	−0.122	−0.030
…is impairing my everyday life	0.273	−0.274	**0.769**	0.101	−0.176	−0.172
…requires substantial financial effort	0.323	−0.101	**0.553**	0.167	−0.201	−0.076
…is annoying	0.172	−0.296	**0.755**	0.014	−0.105	−0.164
…is inconvenient	0.163	−0.215	**0.788**	0.143	−0.118	−0.209
…is not worth it [Ex]	0.167	−0.586	**0.464**	0.007	−0.433	−0.169
**Self-efficacy**						
*Cronbach’s Alpha* = 0.715, CR = 0.800, AVE = 0.502, Mean (SD) = 3.27(0.694)						
Overall, I am capable of undertaking allergy management measures	−0.102	0.308	−0.030	0.017	**0.727**	0.245
If required, I am capable of taking allergy management measures consistently	0.233	0.288	0.015	0.162	**0.634**	0.119
If required, I am capable of taking allergy management measures regularly	−0.169	0.214	−0.214	− 0.139	**0.807**	0.144
If required, I am capable of taking allergy management measures despite difficulties [Ex]	−0.229	0.457	−0.327	−0.140	**0.629**	0.087
I can affect my health-related well-being during the pollen season	−0.231	0.202	−0.224	−0.047	**0.653**	0.016
My health-related well-being during the pollen season is dependent on my health behavior [Ex]	0.099	0.217	−0.181	0.048	0.013	**0.707**
If I take care of my allergy management during the pollen season, I can avoid severe allergic symptom [Ex]	0.025	0.276	−0.016	0.174	0.282	**0.679**
Proper allergy management requires me to change my habits, which is difficult for me [Ex] [R]	0.146	0.162	−0.043	0.181	0.140	**0.664**
My health-related well-being during the pollen season is dependent exclusively on factors I cannot control [Ex][R]	−0.213	0.108	−0.472	−0.136	0.056	**0.538**
If required, I can overcome inconveniences and difficulties related to allergy management during the pollen season [Ex]	−0.253	0.274	−0.320	−0.033	0.219	**0.270**

Note. [Ex] denotes items that were excluded from use in the scales due to their low factor loadings or cross-construct loadings. [R] indicates reverse coded items

The discriminant validity of the measurement model was assessed by a visual examination of the matrix of loadings and cross-loadings of the latent constructs. As shown in Table 3, the items load more on the latent constructs they are supposed to load. Furthermore, the square roots of AVE values for all constructs were larger than the correlations with other constructs (Table 4). Therefore, it can be concluded that the constructs show appropriate discriminant validity.

**Table 4:** Correlations and square roots of average variance extracted

	(1)	(2)	(3)	(4)	(5)
(1) Seriousness	**0.713**				
(2) Severity	0.633	**0.775**			
(3) Response efficacy	0.184	0.025	**0.773**		
(4) Response costs	0.148	0.310	−0.345	**0.726**	
(5) Self-efficacy	−0.136	−0.301	0.600	−0.397	**0.709**

Since the reliability and construct validity conditions were met, the analysis continued with the confirmatory factor analysis (CFA) to test the overall appropriateness of the proposed measurement model. The CFA revealed the *χ*^*2*^*/df* ratio of 1.99, which was slightly below the recommended value of 2.0. The TLI and CFI were 0.917 and 0.934, respectively, representing an appropriate model fit. The RMSE accounted for 0.052, also indicating an appropriate mode fit. Detailed information on the results of the CFA can be found in the supplementary materials. Consequently, the measurement model can be used for inferencestatistical analysis.

### Influencing factors of the utilization of allergy management measures

The results of the average PMT scale scores are shown in Table 3. The perceived seriousness of hay fever and perceived *severity* accounted for 3.53 (SD = 0.786) and 3.62 (SD = 0.848), respectively. Both values were above the mean of the scale, nevertheless, did not indicate threat appraisal to be high. Regarding the response appraisal, the average allergy management measures score was 3.91 (SD = 0.714), indicating high perceived *response efficacy* of allergy management measures*.* The score for the *response costs* scale was 2.95 (SD = 0.882), indicating moderate expected effort associated with allergy management. Consequently, the benefits of hay fever management appeared to outweigh the required efforts. *Self-efficacy* had a score of 3.27 (SD = 0.694), indicating a moderate perceived ability to perform the target behavior.

The correlation coefficients of the average PMT scale scores are shown in Table 4. The perceived *seriousness* of the disease was strongly correlated with the perceived *severit*y of allergic symptoms (ρ = 0.633). The perceived *severity* was also moderately correlated with *response costs* (ρ = 0.310), indicating a higher perceived effort associated with higher symptom severity. The *response costs* showed a moderate negative relationship with *self-efficacy* (ρ = − 0.345) and *response efficacy* (ρ = − 0.397), suggesting that perceptions of one’s own ability to perform the target behavior and perceptions of the efficacy of allergy management decrease with increasing symptom severity. *Response efficacy* was strongly correlated with *self-efficacy,* suggesting that both constructs reinforced each other.

Factors influencing the utilization of various allergy management measures are shown in Table *5*. Three variables were found to exert a positive effect on the willingness to manage hay fever under medical supervision, with *self-efficacy* increasing the probability of treatment under medical supervision almost 5-fold (*OR* = 4.52, 95% CI: 3.11–6.56). Perceived *seriousness* of hay fever had a slightly stronger positive effect on treatment under medical supervision (*OR* = 2.12, 95% CI: 1.56–2.89) than perceived *severity* of symptoms (*OR* = 1.35, 95% CI: 1.02–1.81).

**Table 5:** Association between the PMT constructs and examined allergy management measures

	Odds ratio (95% confidence interval)				
	Model 1 Medical supervision ^a^	Model 2 Self-management^a^	Model 3 Anti-allergic medication^a^	Model 4 Specific immunotherapy ^a^	Model 5 Allergen avoidance^a^
Reference category (Yes ^a^)					
Seriousness	2.12 (1.56–2.89)**	1.04 (0.77–1.41)	0.95 (0.65–1.40)	1.13 (0.81–1.56)	0.72 (0.54–0.96)*†
Severity	1.35 (1.02–1.81)*	1.60 (1.21–2.11)**	1.65 (1.16–2.35)**	0.83 (0.61–1.13)	2.12 (1.60–2.81)**
Response efficacy	1.16 (0.83–1.62)	1.00 (0.73–1.38)	1.40 (0.96–2.06)	1.08 (0.78–1.58)	1.04 (0.77–1.42)
Response costs	1.34 (1.03–1.73)	0.88 (0.67–1.13)	1.09 (0.79–1.51)	1.25 (0.95–1.64)	1.28 (1.00–1.64)
Self-efficacy	4.52 (3.11–6.56)**	1.37 (0.99–1.90)*	1.52 (1.01–2.28)*	0.97 (0.67–1.32)	1.46 (1.07–1.99)*†
Allergy experience	1.00 (0.98–1.03)	1.02 (1.00–1.04)	1.04 (1.00–1.07)*†	1.00 (0.98–1.03)	1.01 (0.99–1.03)
Gender (female)	1.17 (0.79–1.63)	1.44 (1.25–2.26)*†	1.25 (0.96–2.17)	0.70 (0.45–1.11)	1.03 (0.81–1.69)
Medical supervision ^a^		0.39 (0.25–0.86)**	2.22 (1.48–3.35)*	9.80 (8.16–13.80)**	0.66 (0.44–1.09)
Self-management ^a^			4.76 (2.67–7.49)**	0.91 (0.59–1.51)	2.56 (1.87–3.52)**
Anti-allergic medication ^a^				1.26 (0.66–2.40)	1.17 (0.67–1.87)
Specific immunotherapy ^a^					1.09 (0.67–1.78)

*Significance level *p* < 0.05, ** Significance level *p* < 0.01, † not significant due to multiple testing, ^a^ Reference category “taking action”

Model 1: R^2^ = 0.29 (Cox & Snell), 0.37 (Nagelkerke), χ^2^ (7) 169.74, *p* < 0.00; Model 2: R^2^ = 0.25 (Cox & Snell), 0.33 (Nagelkerke), χ^2^ (8) 168.24, *p* < 0.00; Model 3: R^2^ = 0.47 (Cox & Snell), 0.62 (Nagelkerke), χ^2^ (9) 347.43, *p* < 0.00; Model 4: R^2^ = 0.37 (Cox & Snell), 0.49 (Nagelkerke), χ^2^ (10) 250.47, *p* < 0.00; Model 5: R^2^ = 0.21 (Cox & Snell), 0.28 (Nagelkerke), χ^2^ (11) 121.75, *p* < 0.00

Perceived symptom *severity* showed a significant positive effect on the decision to manage hay fever without medical support, increasing its probability by 60% (*OR* = 1.60, 95% CI: 1.21–2.11). Females (*OR* = 1.44, 95% CI: 1.25–2.26) were significantly more likely to self-manage their hay fever than men, but this finding was rejected due to multiple testing. Participants who reported being treated by a doctor were significantly less likely to perform additional self-management measures (*OR* = 0.39, CI: 0.25–0.86). This finding suggests that allergic individuals opt for one of the two alternatives, either hay fever self-management or hay fever treatment under medical supervision. However, these groups are still not completely distinct.

*Self-efficacy* (*OR* = 1.52, 95% CI: 1.01–2.28) and perceived symptom *severity* (*OR* = 1.65, 95% CI: 1.16–2.35) exerted positive effects on the decision to take anti-allergic medication, with the latter construct being a slightly stronger predictor. Hay fever management under medical supervision and hay fever self-management had even stronger impacts on the likelihood of taking anti-allergic medication. Those receiving medical help were twice as likely to take antihistamines (*OR* = 2.22, 95% CI: 1.48–3.35), whereas allergic individuals self-managing their hay fever were 4.5 times more likely to utilize anti-allergic medication (*OR* = 4.48, 95% CI: 2.67–7.49).

Only one variable predicted the decision to undergo specific immunotherapy. Participants being treated by a doctor were 10 times as likely to make use of this curative therapy option (*OR* = 9.80, 95% CI: 8.16–13.80).

Perceived symptom *severity* (*OR* = 2.12, 95% CI: 1.60–2.81) and *self-efficacy* (*OR* = 1.46, 95% CI: 1.07–1.99) increased the likelihood of allergen avoidance, whereas increasing perceived *seriousness* of the disease was associated with decreasing willingness to undertake avoidance strategies (*OR* = 0.72, 95% CI: 0.54–0.96). However, the latter finding was found to be nonsignificant after multiple testing. Nevertheless, allergic individuals preferring hay fever self-management were significantly less reluctant to make use of allergen avoidance strategies (*OR* = 2.56, 95% CI: 1.87–3.52).

## Discussion

The presented empirical investigation has revealed three major findings and makes the following key contributions. First, health-related decisions regarding the utilization of various hay fever management measures were substantially stronger motivated by threat appraisal than by response appraisal. Second, the perceived *severity* of symptoms was the dominant driver of threat appraisal, which facilitated health-related decisions. Third, *self-efficacy* was the only significant influencing factor of response appraisal affecting hay fever management decisions in allergic individuals.

In the consideration of these survey results, it must be noted that participation in this survey was voluntary, and the online questionnaire was also distributed to all students of the University of Augsburg. Thus, a large proportion of the study participants were young, well-educated allergic individuals. In this way, there is a certain degree of bias in the respondents’ answers, as the study sample might represent a specific population. However, young adults who are students exhibit a higher prevalence of allergic sensitization than other age groups [40]. The possible bias was minimized through thoughtful and careful scale development based on interviews and a pretest with a sample from the target population. Furthermore, our analysis relied on participants’ self-reported information concerning the utilization of different hay fever management measures and suffering from hay fever in general. The main limitation of the present survey was the relatively small sample size, which did not allow us to separately analyze the PMT influencing variables for each TTM stage.

Considering the assessed TTM stages, for each hay fever management measure, the largest share of participants was in either the pre-contemplation or maintenance stage. This finding indicates that most allergic individuals either had utilized an allergy management measure for a long time or did not even intend to it. Considering that the mean duration of allergies was almost 15 years, the included allergic individuals might have developed well-established habits concerning their approach to managing hay fever, especially if their existing behavior was perceived as reasonable and satisfactory. The only exception was found regarding the utilization of specific immunotherapy, for which the group of *nonactors* was larger than the group of *actors*. Furthermore, the largest portion of allergic individuals in the maintenance stage was found for the intake of anti-allergic medication, and the largest proportion of allergic individuals in the pre-contemplation stage was found for specific immunotherapy. The smallest share of allergic individuals was in the preparation stage across all questioned hay fever management measures except for specific immunotherapy. This finding suggests that individuals either quickly translate their intentions into action or fall back into the contemplation stage. A similar finding was reported by Schwarzer (1999), who emphasized that holding a strong intention does not guarantee the initiation of action. People may fail to address self-regulatory problems during behavior change due to various obstacles, such as changes in the surrounding context [41].

Three PMT constructs were shown to significantly influence health-related decisions concerning hay fever management. First, the perceived *seriousness* of hay fever had a significant positive effect on the decision to seek medical support and tended to be negatively related to allergen avoidance strategies. Second, perceived hay fever *severity* showed a significant positive effect on four allergy management measures, including treatment under medical supervision, allergy self-management, intake of anti-allergic medication, and allergen avoidance. Third, *self-efficacy* was found to be a significant predictor for three health-related measures, namely, treatment under medical supervision, intake of anti-allergic medication, and allergen avoidance. Additionally, *self-efficacy* tended to be positively related to allergy self-management. Interestingly, perceived *self-efficacy* had a substantially stronger effect on the decision to treat allergy under medical supervision than to practice allergy self-management. Remarkably, neither *response efficacy* nor *response costs* significantly influenced health-related decisions in hay fever management.

From the insights presented above, two conclusions can be drawn. First, since perceived symptom *severity* positively influenced all except one investigated allergy management measure, occurring allergic symptoms might be considered a trigger that induces action for allergy management. This conclusion is consistent with the results of Meltzer et al. (2017), who showed hay fever sufferers to consider the onset of allergic symptoms to be a starting point for their medication [8]. Second, anti-allergic measures are induced rather by threat appraisal than response appraisal. This observation is in line with the results of Ferrer and Klein (2015), who showed that health-related risk perceptions appear to play a more important role in motivating behavior change than perceived *response efficacy* [42]. Furthermore, this finding supports the so-called early-effectiveness hypothesis, which suggests the fear appeal to be more effective in prompting people to change behavior because they initially need to understand that a threat exists to develop motivation and increase their commitment to adopt new behavior [43].

In addition to the influence of the PMT constructs, several allergy management measures were significantly related to each other. In particular, participants who engaged in allergy self-management were more likely to take anti-allergic medication, whereas allergic individuals being supported by a doctor in hay fever management were not. Two possible reasons might be responsible for these findings. First, the intake of medicine is the most obvious measure to address some health-related issues. Second, hay fever sufferers can buy several over-the-counter antihistamines without medical advice or prescription, which makes this allergy management option popular among hay fever sufferers [44, 45]. The use of specific immunotherapy, as the only curative treatment option, was significantly predicted only by medical supervision. On the one hand, this finding is self-explanatory because specific immunotherapy can be carried out exclusively under medical supervision. On the other hand, it remains unclear why none of the PMT constructs significantly influenced the decision to undergo specific immunotherapy. We suggest that allergic individuals seeking medical supervision completely rely on doctors’ advice without considering further factors.

Positive, informed changes in health-related behavior are a desirable endpoint for the problem of inadequate allergy management. Educational interventions providing general knowledge about diseases and possible self-management strategies have been shown to have a beneficial effect on behavioral change and health outcomes in chronically ill individuals [46–48]. Vulnerable individuals suffering from chronic health conditions are interested in receiving information on protective behavior related to their disease, especially regarding immediate-term advice on health management [49]. Concrete advice on health behavior during the pollen season might be beneficial for hay fever sufferers since even general health recommendations such as the importance of adequate sleep duration and appropriate body weight might be relevant to decrease the risk of suffering allergic symptoms [50]. Based on the insights of the present study, health education for allergic individuals should be focused on variables related to threat appraisal, especially on the perceived *seriousness* of the disease. The manipulation of threat appraisal variables was shown to significantly change perceptions of disease and, consequently, the health behavior of people concerned [51]. However, it has to be taken into account that fear appeals have the potential to promote maladaptive responses, such as defensive psychological tactics to resist negative messages [52]. Particularly, if the threat appraisal is high and the possible response to threat is perceived to be ineffective. Fear appeals accompanied by increasing *self-efficacy* and *response efficacy* and decreasing *response costs* appear to be more effective [53]. Information on coping that is aimed at increasing perceived *response efficacy* perceived *self-efficacy* is more effective in enhancing protective intention than health information that only increases the perceived threat [54]. Since all motivators are rooted in the core belief that one has the power to achieve the desired change, the second focus of health education should be on increasing *self-efficacy* in allergic individuals. Indeed, promoting more positive attitudes can facilitate behavior change by increasing *self-efficacy* for healthier behavior [55].

## Conclusion

According to the insights offered by this study, a large share of allergic individuals do not engage in allergy management under medical supervision and tend to treat themselves self-reliantly. Since a large share of allergic individuals uses over-the-counter medication, obtaining first-line advice from a pharmacist might be considered a valuable alternative to medical supervision if the latter is not available for any reason. A promising possibility to facilitate health behavior among hay fever sufferers is the implementation of educational interventions aimed at helping individuals evaluate their existing hay fever management routines and obtain additional information on allergy management. Educational interventions to improve allergy management should focus on increasing awareness of health-related risks associated with allergic diseases for individuals with unappropriated allergy management. If such interventions are accompanied by the provision of information on various instrumental and psychological coping strategies to increase the perceived self-efficacy of allergic individuals, an even stronger effect on the desired target behavior might be achieved.

## Supplementary Information

**Additional file 1.** Operationalization of the PMT constructs.

## Data Availability

The datasets used and/or analyzed during the current study are available from the corresponding author on reasonable request.

## Abbreviations

PMT: Protection motivation theory
TTM: Transtheoretical model
