# Supplementary material for: Determinants of the utilization of allergy management measures among hay fever sufferers: a theory-based cross-sectional study
Source: BMC Public Health. 2020 Dec 7;20:1876. doi: 10.1186/s12889-020-09959-w (PMC7720499; doi:10.1186/s12889-020-09959-w)
Supplement: Supplementary file 1 — Additional file 1. Operationalization of the PMT constructs. [file 12889_2020_9959_MOESM1_ESM.docx]

**Supplementary materials**

**Semistructured interview guide**

**Introduction:**

- Greetings and self-introduction of the interviewer
- Explanation of the objective of the project and content of today’s conversation
- Confidentiality statement
- Anonymity statement

**Opening:**

- How are you doing today?

**Demographics:**

- How old are you?
- To which pollen species are you allergic?
- For how many years have you suffered from hay fever?
- Which measures do you undertake to combat your hay fever?

**Chronicity of hay fever**

- In your opinion, is hay fever a disease which has to be treated/managed? [yes/no]
- Provide some arguments in favor of or against the necessity of hay fever management/treatment.
- What is your understanding of a chronic disease?
- Do you perceive your hay fever to be a chronic disease? [yes/no]
- Which characteristics of your hay fever suggest that it is a chronic disease?
- Can you think of some characteristics of your hay fever that argue against its chronicity?

**Severity of hay fever**

- On a scale from 1 to 10, how severe are your allergic symptoms while you are symptomatic?
- Does hay fever have an impact on your health-related well-being while you are symptomatic? [yes/no]
- Can you specify which dimensions of your life are negatively affected by allergic symptoms?
- What are the manifestations of the negative effects caused by allergic symptoms in [mentioned dimension]?
  - Physical well-being
  - Everyday routines
  - Social functioning
  - Workplace productivity
  - Sleep quality
  - Psychological well-being

**Response efficacy**

- What is your definition of good/efficient disease treatment/management?
- Which effect do you expect from anti-allergic measures?
- Which effect should be a certain allergy management measure capable of to be considered beneficial?

[If not mentioned, ask for following dimensions]

- - Complete symptom relief/Weakening existing symptoms
  - Prevention of asthma
  - Prevention of symptom worsening
  - Improved control over symptoms
  - Improvement in which dimensions of your everyday life?
- Do you treat/manage your hay fever? [yes/no]
- Which of the mentioned effects have you experienced due to undertaking anti-allergy measures? [Refer to the dimensions mentioned as being negatively affected by allergic symptoms]

**Response costs**

- Do you experience inconveniences due to undertaking anti-allergy measures? [yes/no] Can you specify which exactly?
- Which dimension of your everyday life is affected by this inconvenience?
- Do you experience difficulties related to allergy management? What are they?

[If not mentioned, ask about the following dimensions]

- - Time effort
  - Financial effort
  - Lack of knowledge
  - Lack of further resources
  - Social embarrassment
  - Need to change routines every day
- Do you see some disadvantages related to the need to perform allergy management?

**Self-efficacy**

- What is important to make long-term treatment/disease management work?
- From your point of view, are you capable of changing your health-related well-being while symptomatic? To what extent?
- What is important to make allergy management effective?
- Which personal traits do you think are important for managing hay fever properly? Which personal traits facilitate hay fever management?
- Are you capable of managing your hay fever yourself?

**Final questionnaire**

[Five-step Likert scale: *“totally disagree”, “rather disagree”, “neutral”, “rather agree”, “totally agree”*]

**Seriousness**

- Hay fever is a disease
- Hay fever is a chronic health condition
- Hay fever is a serious health condition
- Hay fever influences an individual’s well-being
- I see my hay fever as a long-term health condition
- Hay fever is a health condition that has to be treated

**Severity**

Due to symptoms of my hay fever…

- …I feel physically burdened
- …I feel limited in my social life
- …I feel limited in my spare time
- …my sleep quality is burdened
- …my overall well-being is burdened
- …I experience a loss of productivity

**Response efficacy**

Allergy management*…

*[*All medical and non-medical measures aiming in preventing or weakening symptoms caused by airborne allergens]*

- …weakens symptom severity
- …helps better control allergic symptoms
- …helps manage everyday routines
- …improves overall well-being
- …helps in staying productive during the pollen season

**Response costs**

Allergy management*…

*[*All medical and non-medical measures aiming in preventing or weakening symptoms caused by airborne allergens]*

- …is time consuming
- …is impairing my everyday life
- …requires substantial financial effort
- …is annoying
- …is inconvenient
- …is not worth it

**Self-efficacy**

*[*All medical and non-medical measures aiming in preventing or weakening symptoms caused by airborne allergens]*

- Overall, I am capable of undertaking allergy management measures
- If required, I am capable of taking allergy management measures consistently
- If required, I am capable of taking allergy management measures regularly
- If required, I am capable of taking allergy management measures to overcome difficulties
- I can affect my health-related well-being during the pollen season
- My health-related well-being during the pollen season is dependent on my health behavior
- If I take care of my allergy management during the pollen season, I can avoid severe allergic symptoms
- Proper allergy management requires me to change my habits, which is difficult for me
- My health-related well-being during the pollen season is dependent exclusively on factors I cannot control
- If required, I can overcome inconveniences and difficulties related to allergy management during the pollen season

**Demographics**

- Please, state your gender.
- For how many years are you suffering from allergy?
- To which allergens are you sensitized?
- How strong are your allergic symptoms?
- In which months do allergic symptoms occur?

**Allergy management**

*[*Five-step Likert scale: *“no, and I am not intend to”, “no, but I might think about it”, “no, but I am strongly intended to do so”, “yes, but I am doing it recently”, “yes, and I am doing it for a long time”]*

- I treat my allergic symptoms under medical supervision
- I manage my allergic symptoms self-reliantly
- I take anti-allergic medication
- I undergo specific immunotherapy

I adapt my behavior inside a pollen season in order to avoid exposure to pollen

**Result of the confirmatory factor analysis**

-.397

*Model: χ^2^ (199) = 397.57, CFI =0.934, TLI = 0.917, GFI = 0.956, RMSEA = 0.052*

SER

SEV

RE

RC

SE

.58

.72

.67

.64

.70

.68

.47

.89

.57

.778

.80

.64

.68

.72

.76

.60

.79

.75

.79

.59

.72

.60

SER1

SER2

SER3

SEV1

SEV2

SER3

SER4

SER5

SER6

RE1

RE2

RE3

RE4

RC1

RC2

RC3

RC4

RC5

SE1

SE2

SE3

SE4

-.136

-.301

.148

.600

.310

.184

-.345

.025

.633
